# Supplementary material for: Chemical Genetic Targeting of the LRRK2 GTPase Domain
Source: bioRxiv. 2026 Sep 7:2026.09.03.749217. Preprint. [Version 1] doi: 10.64898/2026.09.03.749217 (PMC13622542; doi:10.64898/2026.09.03.749217)
Supplement: Supplementary file 1 [file media-1.pdf]

Supplementary Figures

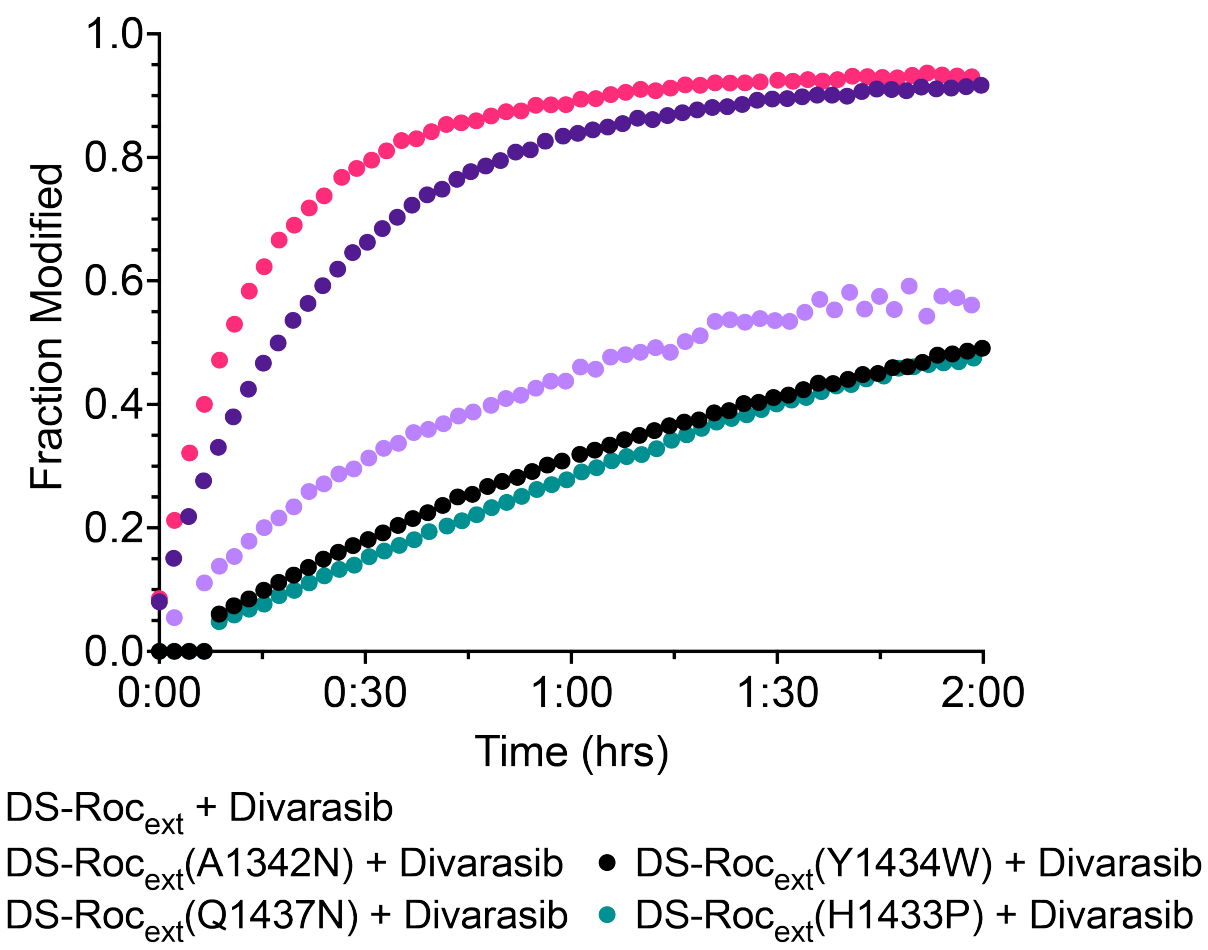

Supplementary Figure 1: Reversion of individual drug-sensitizing mutations diminishes divarasib covalent labeling of DS-Roc$_{ext}$.

a.

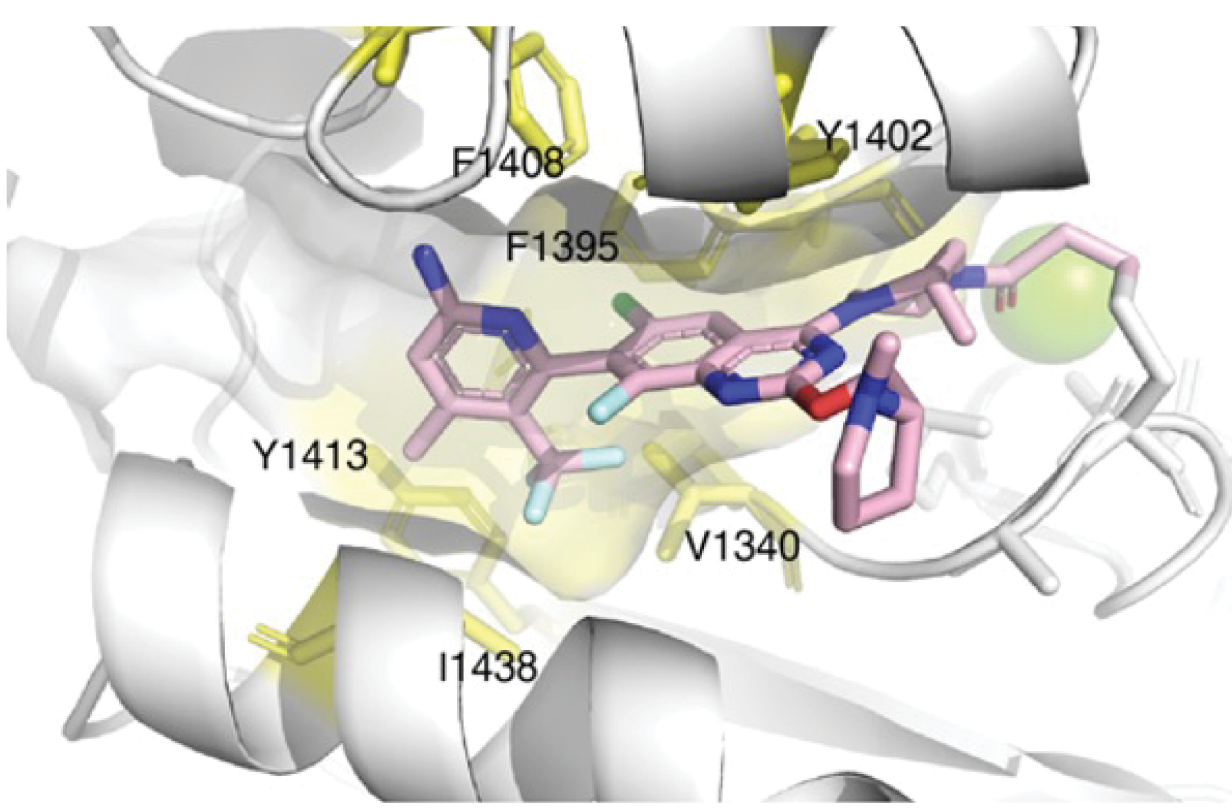

b.

WT	5Xv2	WT + GDP	5Xv2 + GDP	WT + GTPγS	5Xv2 + GTPγS
22.54	24.37	47.83	47.31	35.53	28.41

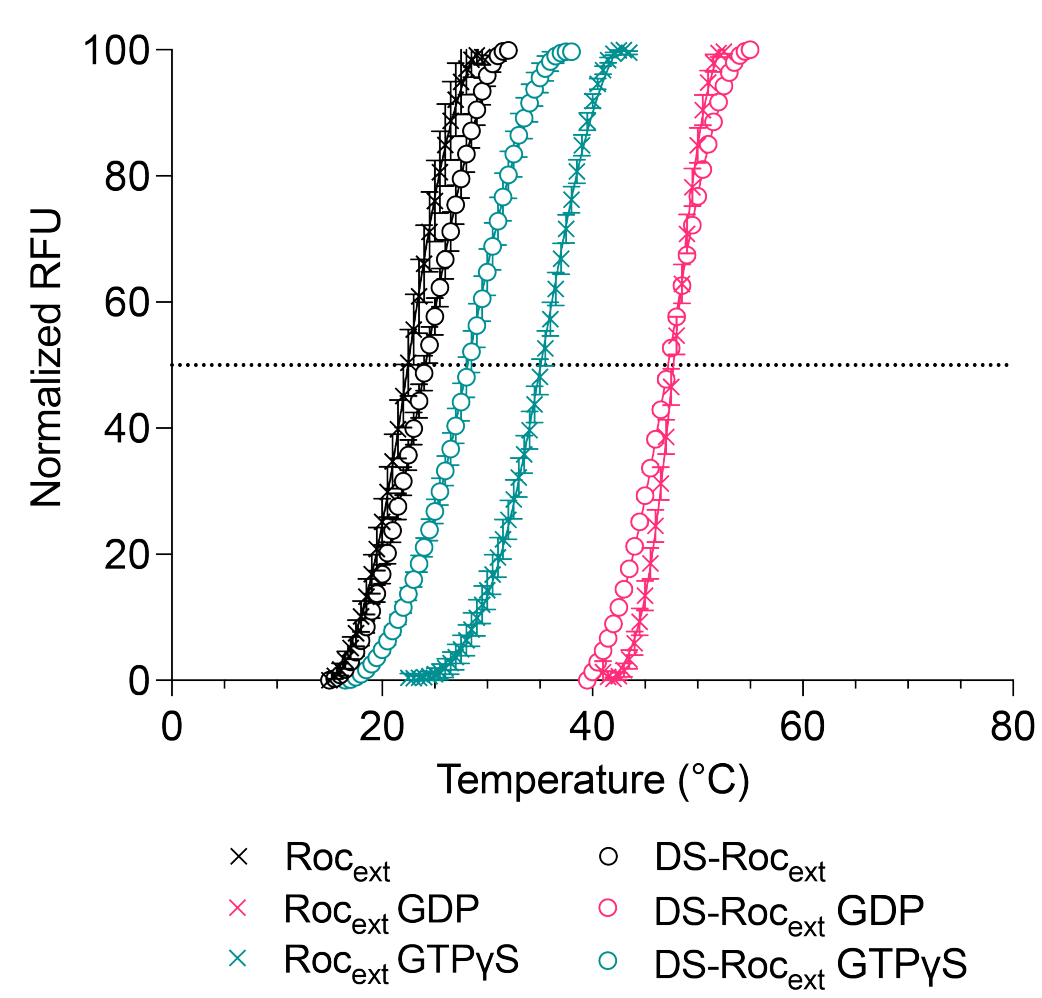

Supplementary Figure 2: A) CryoEM structure of DS-Roc$_{ext}$ with represented native residues (yellow) contributing to the deep hydrophobic pocket SII pocket. **B)** Comparison of WT-Roc$_{ext}$ and DS-Roc$_{ext}$ DSF performed in nucleotide-saturating conditions.

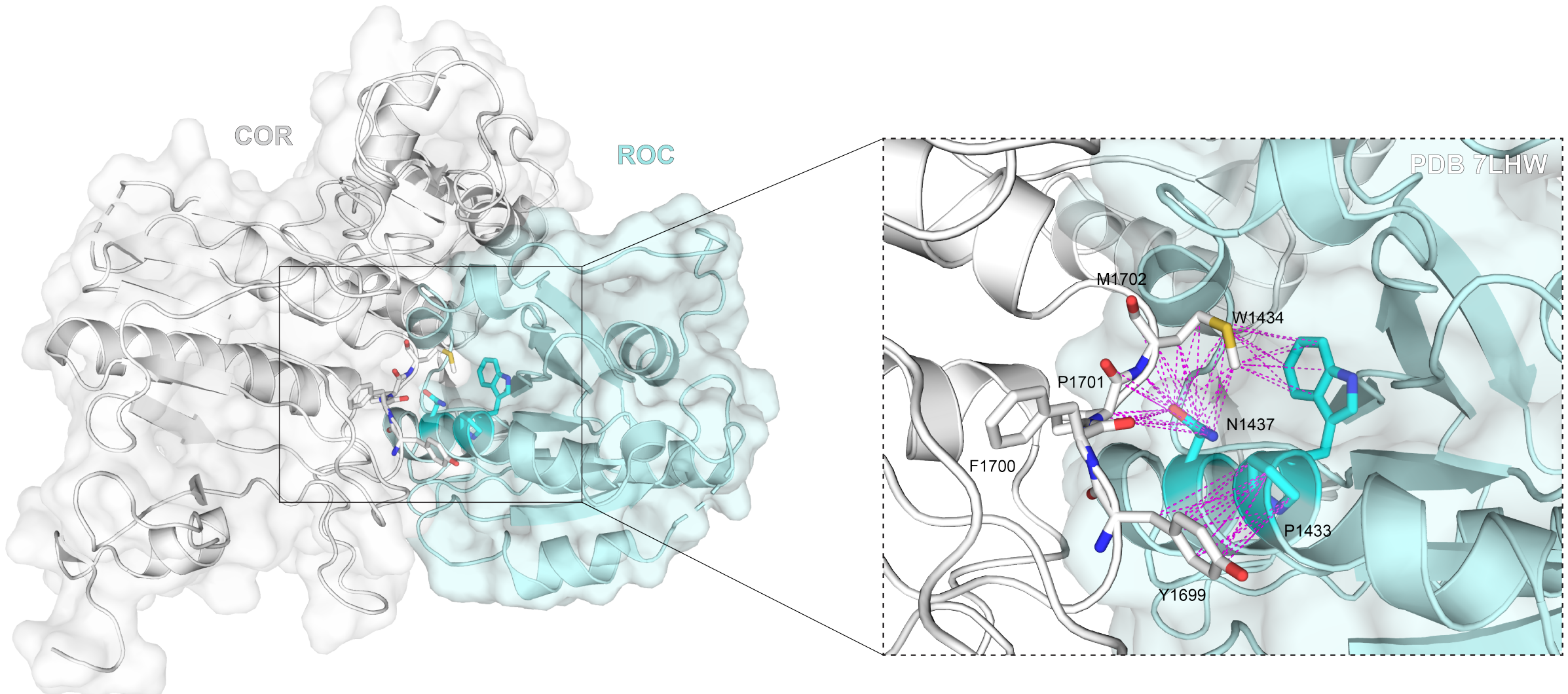

Supplementary Figure 3: Drug-sensitizing mutations on the α3 helix contact COR-B residues within a 5Å distance.

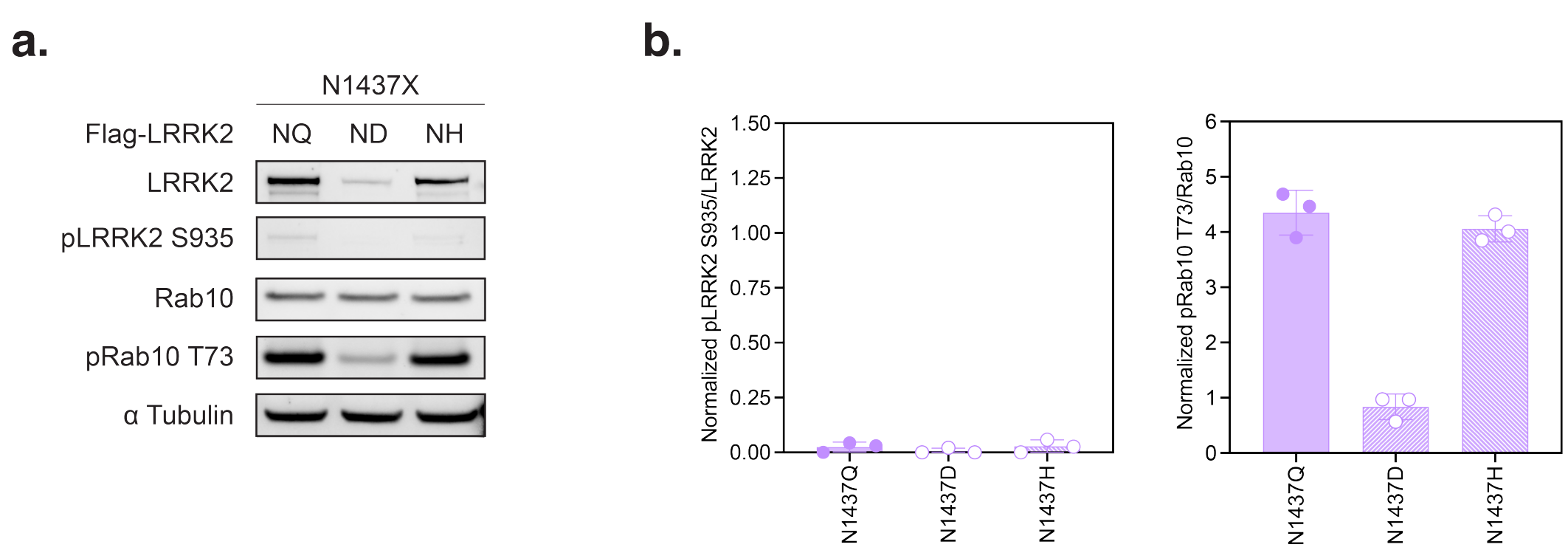

Supplementary Figure 4: Drug-sensitizing mutation N1437Q elevates kinase activity in DS-LRRK2. **A)** Immunoblot analysis of HEK293T cells overexpressing FLAG-LRRK2 containing mutation N1437Q (NQ), N1437D (ND), or N1437H (NH). **B)** Immunoblot quantification of LRRK2 S935 phosphorylation and Rab10 T73 phosphorylation for immunoblot shown in Supplementary Figure 4A. Immunoblot quantifications are mean +/- SD (n=3 independent experiments).

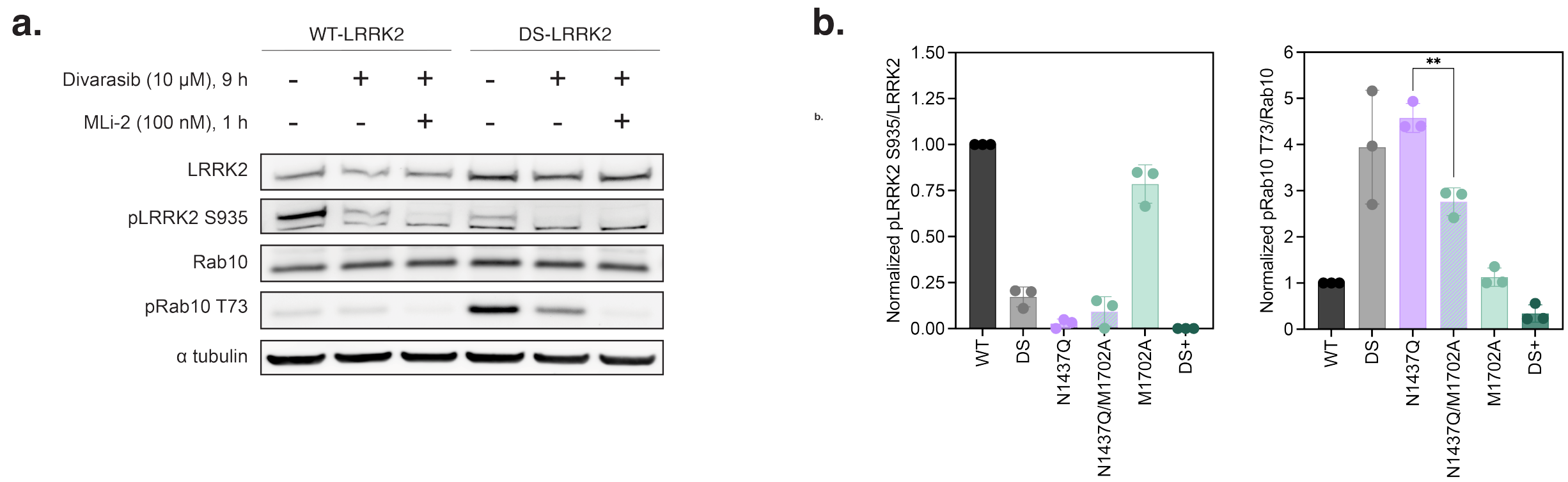

Supplementary Figure 5: **A)** Immunoblot analysis of HEK293T cells overexpressing WT-FLAG-LRRK2 or DS-FLAG-LRRK2 sequentially treated with 10 µM Divarasib for 8 hours then 100 nM MLi-2 for 1 hour. **B)** Immunoblot quantification of LRRK2 S935 phosphorylation and Rab10 T73 phosphorylation for immunoblot shown in Figure 4E. Immunoblot quantifications are mean +/- SD (n=3 independent experiments).

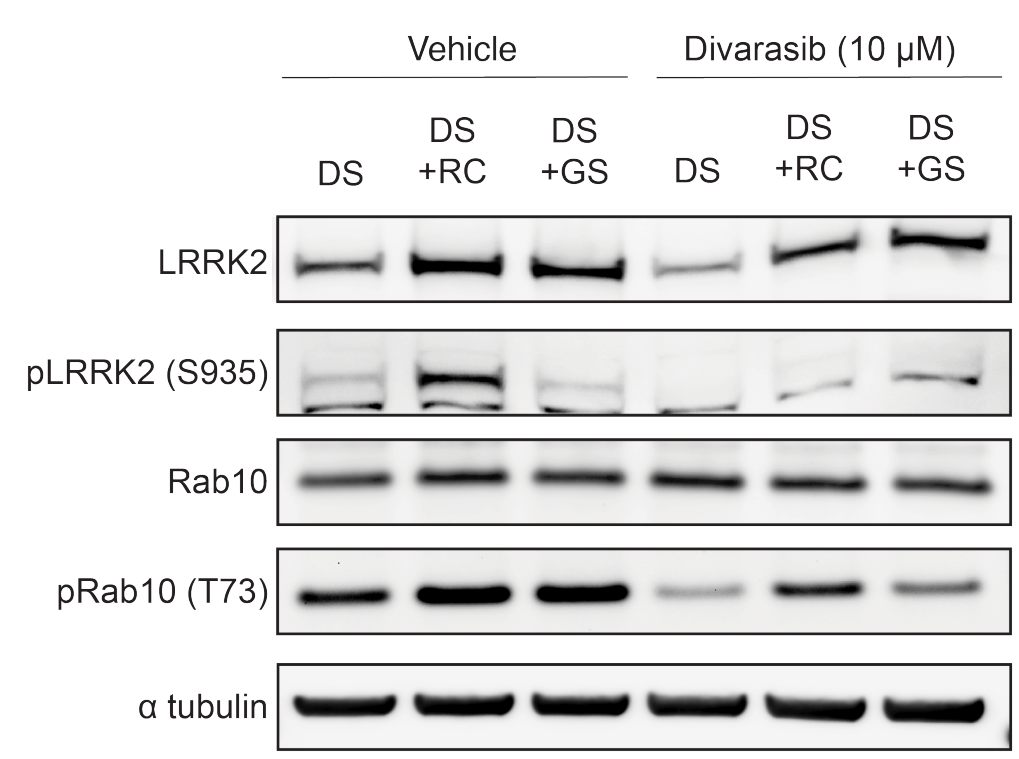

Supplementary Figure 6. Immunoblot analysis of HEK293T cells overexpressing DS-FLAG-LRRK2, DS-FLAG-LRRK2 with R1441C (DS+RC), or DS-FLAG-LRRK2 with G2019S (DS+GS) treated with 10 µM divarasib for 8 hours.

Supplementary Table 1 Crystallographic data collection and refinement statistics.

Parameter	Value
**Data Collection**	
Space group	$P2_12_12_1$
Unit cell dimensions	
a, b, c (Å)	40.41, 98.38, 105.20
α, β, γ (°)	90.00, 90.00, 90.00
Resolution range (Å)*	49.19–2.10 (2.16–2.10)
R^*_{merge}	0.319 (2.227)
$\text{CC}^*_{1/2}$	0.977 (0.375)
$\langle I/\sigma(I)\rangle^*$	7.9 (1.2)
Completeness (%)*	99.8 (100.0)
Redundancy / Multiplicity*	6.2 (6.4)
**Refinement**	
Resolution range (Å)	35.22–2.30
No. reflections (work / test)	17,332 / 1,935
$R_{\text{work}}/R_{\text{free}}$	0.2255 / 0.2616
Wilson B-factor	41.59
Overall mean	52.32
Protein (Chain A / Chain B)	61.77 / 44.01 (Mean: 52.36)
Ligand (GDP / LRJ)	58.27 / 53.43 (Chain D: 65.79, Chain G: 41.06)
Solvent (Water, 101 atoms)	46.95
Bond lengths (Å)	0.002
Bond angles (°)	0.580
Clashscore	5.87
**Ramachandran analysis**	
Favored (%)	98.48
Allowed (%)	1.52
Outliers (%)	0.00
Rotamer outliers (%)	1.49
C_β deviations (%)	0.00
Coordinate error (ML-based, Å)	0.32

Supplementary Table 2 Tm comparison of WT-Roc_{ext} and DS-Roc_{ext} in nucleotide-saturating conditions. Temperatures listed in ˚C.

	WT-Roc_{ext}	DS-Roc_{ext}
No nucleotide	22.54	24.37
GDP	47.83	47.31
GTPγS	35.53	28.41
